# The Presence of a Large Chiari Network in a Patient with Atrial Fibrillation and Stroke

**DOI:** 10.1155/2016/4839315

**Published:** 2016-07-31

**Authors:** Nneka Schwimmer-Okike, Johannes Niebuhr, Grit Gesine Ruth Schramek, Stefan Frantz, Heike Kielstein

**Affiliations:** ^1^Department of Anatomy and Cell Biology, Faculty of Medicine, Martin Luther University Halle-Wittenberg, 06108 Halle (Saale), Germany; ^2^Institute for Pathology, University Hospital Halle, 06108 Halle (Saale), Germany; ^3^Department of Internal Medicine III, University Hospital Halle, 06120 Halle (Saale), Germany

## Abstract

The Chiari network is an embryological remnant found in the right atrium, mostly without any significant pathophysiological consequences. However, several cardiac associations are reported in the literature including supraventricular tachyarrhythmias. We present a case of a 96-year-old body donor with a stroke episode and intermittent atrial fibrillations. The dissection of the heart revealed the presence of an immense Chiari network with a large central thrombus. The role of a Chiari network in the pathogenesis of stroke and pulmonary embolism is discussed.

## 1. Introduction

While the fetal heart is developing the venous confluence in the right atrium has two valves, called Eustachii and Thebesii. Both valves are secured in the right atrium by the septum spurium. Later in the development these valves and the septum spurium usually regress. However, in some cases the regression remains incomplete resulting in a cor triatriatum. In cases of a partial regression, a fenestrated membrane remains, the so-called Chiari network [1]. While Carl von Rokitansky is believed to be the first describing this anatomical remnant in a single case, it was named after Hans Chiari who described it in a review of 11 cases [2]. In the beginning the Chiari network was believed to be clinically silent, but several publications showed a possible link to cardiac diseases like embolism [3], infective endocarditis [4], or supraventricular tachyarrhythmias [5]. In the vast majority of published cases the Chiari network was diagnosed and illustrated by echocardiography. Here, we report a case of a Chiari network with prior atrial fibrillation and stroke. The immense Chiari network is shown in the dissected heart of the patient.

## 2. Case Report

A 96-year-old man was hospitalized because of a neurological failure. The patient showed a flaccid paralysis of the right arm and leg, an incomplete facial paresis on the right, dysphagia, global aphasia, and unilateral neglect, suggesting a stroke. The cranial CT showed no signs of bleeding and no middle line shift. Electrocardiography revealed a tachyarrhythmia caused by an atrial fibrillation with a rate of 100–140 beats/min. Patients' medical record showed intermitted atrial fibrillations and a thrombectomy due to a TIA (transient ischemic attack) six years ahead of the current event. A treated prostate cancer and nontreated squamous cell cancer were further diagnoses of the patient. One month later and still under observation and treatment the patient died in the hospital.

**Figure 1:**
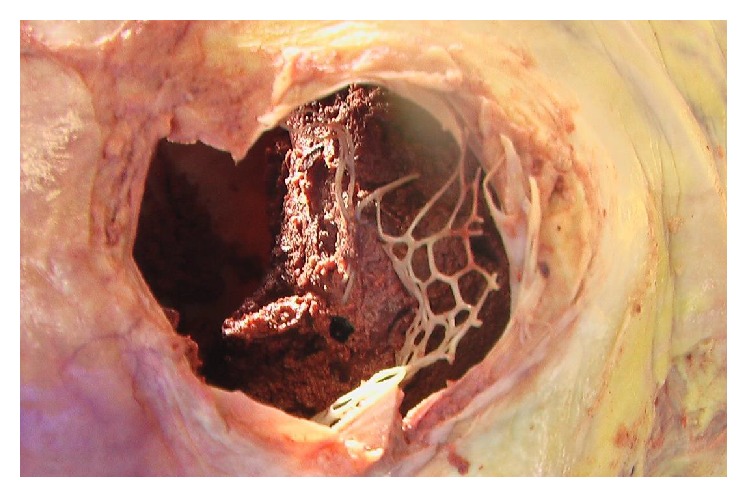
Large thrombus in the center of the Chiari network within the right atrium of the heart.

**Figure 2:**
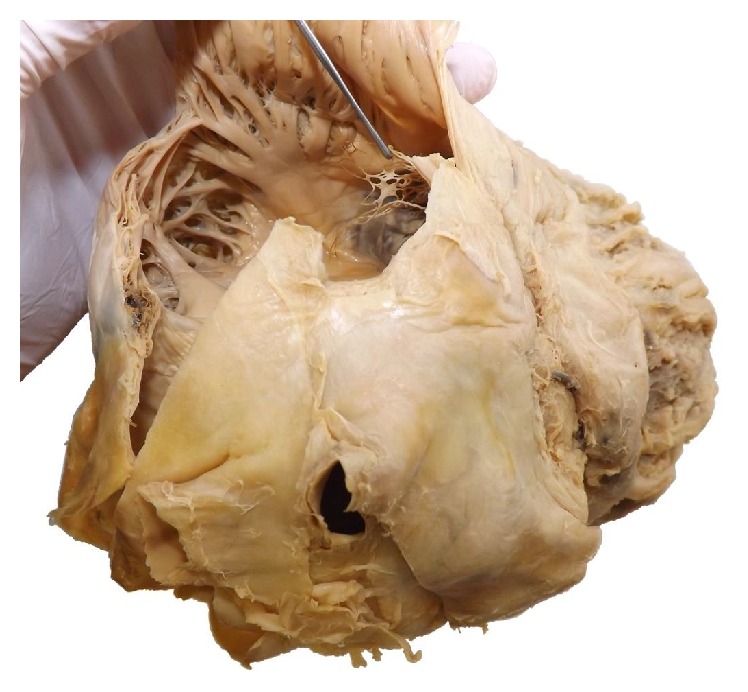
Opened right atrium of the heart and an overview of the Chiari network.

**Figure 3:**
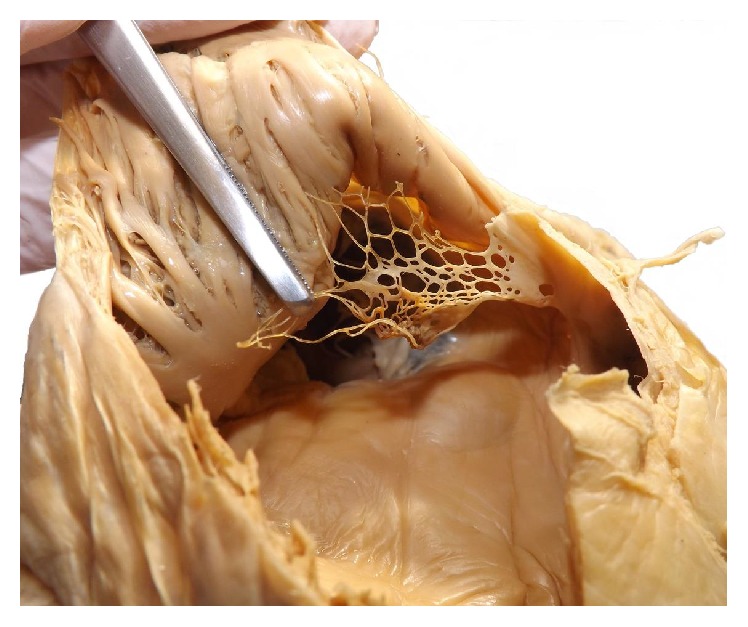
Fixed Chiari network in proximity to the inferior vena cava after removal of the thrombus.

According to his living will the deceased became a body donor for the dissection course at our Institute for Anatomy. During the examination and dissection of the heart the students discovered a net-like structure with a large central thrombus inside the right atrium in close proximity to the inferior vena cava.  Figure 1 shows the orifice of the inferior vena cava with view to the thrombus. After the removal of the thrombus a careful examination of the net structure was performed showing its fixation in the inner surface of the right atrium. The histological examination of the resected net structure showed mainly fibrous connective tissue and no cardiomyocytes. The microscopic and macroscopic aspects are consistent with the Chiari network. Figures 2 and 3 show an overview and the opened right atrium and the immense size of the net (4.0 × 2.5 cm). Because of the stiffness of the tissue caused by the fixation procedure of the body donor we neither could confirm nor ruled out further heart malformations like a patent foramen ovale.

## 3. Discussion

The Chiari network is an anomaly of the heart with a prevalence ranging from 1.3 to 4.0% [6]. However, more recent studies show prevalence rates up to 10.5% [7]. This anatomic variation and embryologic remnant is believed to be mostly clinical inapparent. Nevertheless, several cases have been described showing relevant pathophysiological consequences. Chang described a case of tricuspid valve regurgitation caused by a Chiari network [8] and Goedde and coworkers showed a case of supraventricular fibrillations demanding the surgical removal of the Chiari network [9]. Atrial fibrillation and tricuspid valve regurgitation are relevant diseases and therefore the presence of a Chiari network has to be taken seriously.

The case described by Goedde and coworkers has various similarities with the case presented here: both patients had a Chiari network with a thrombus enmeshed in between. The medical records of both patients showed atrial fibrillations and atrial embolism. While the patient in the case report from Goedde and coworkers suffered from a pulmonary embolism, the patient in the present case had a stroke.

The presence of a patent foramen ovale is a prerequisite to cause a paradoxical embolism, leading to a stroke or pulmonary embolism. Schneider and coworkers demonstrated significantly higher prevalence rates for a patent foramen ovale in patients with a Chiari network and herewith a serious risk for the development of an atrial embolism [10]. However, other studies showed a rather minor role of the presence of a patent foramen ovale in the pathogenesis of paradoxical embolism [11]. Various cases of patients suffering a paradoxical embolism without patent foramen ovale have been published [12, 13].

To date, only few photographs of a Chiari network have been published. Here we show an immense Chiari network as the possible cause for the longtime intermittent atrial fibrillation, a TIA, and a stroke, finally leading to the death of the patient. In conclusion, the possibility of the presence of a Chiari network with or without a patent foramen ovale should be taken into consideration as a possible (additional) diagnosis for a patient suffering from embolism, infective endocarditis, or supraventricular arrthythmias.
